# High prevalence of antibiotic resistance of *Streptococcus* species in saliva from non-hospitalized adults – a pilot study

**DOI:** 10.1080/20002297.2025.2486647

**Published:** 2025-04-02

**Authors:** Maria Nordholt Dollas, Martin Nilsson, Tove Larsen, Nikoline Nygaard, Claus Moser, Daniel Belstrøm

**Affiliations:** a Department of Odontology, Section for Clinical Oral Microbiology, Faculty of Health and Medical Sciences, University of Copenhagen, Copenhagen, Denmark; b Department of Clinical Microbiology, Copenhagen University Hospital, Rigshospitalet, Copenhagen, Denmark; c Department of Immunology and Microbiology, University of Copenhagen, Copenhagen, Denmark

**Keywords:** Antibiotic resistance, saliva, oral microbiome, streptococci, screening

## Abstract

**Background:**

Antibiotic resistance (AR) is a recognized threat to global human health. However, the prevalence of AR in healthy adults is not well described. The present observational pilot study aimed to uncover the potential of using saliva samples for screening for antibiotic resistance.

**Methodology:**

A laboratory protocol was developed for screening of AR in saliva samples, which was tested and validated using saliva samples collected from 100 study participants. The risk of AR was analyzed with descriptive statistics and evaluated using a risk-factor profile based on information on antibiotic usage within the last 12 months, education level and origin of birth.

**Results:**

AR was identified in 43 (48%) saliva samples, out of which 60,0% and 17,1% of resistant strains displayed resistance to clindamycin and penicillin, respectively. Streptococcus salivarius and Streptococcus parasanguinis were most often identified with AR (51,4% of all cases). The risk of AR was not associated with self-perceived oral or general health, antibiotic use within the latest 12 months or any demographic or socioeconomic parameters recorded. The risk-factor profile was observed in 44% in the AR group versus 30% in the non-AR group (*p* = 0.19).

**Conclusion:**

The present study showed that it is possible to perform non-invasive saliva-based screening for AR with a frequency of 48% of the samples, highlighting that saliva samples could be a valuable supplement to current surveillance methodologies for AR in the oral microbiota.

## Introduction

Since the discovery of penicillin in 1929, antibiotics have been instrumental in the treatment of bacterial infections [1]. However, the growing problem of antibiotic resistance (AR) threatens to undermine the benefits of antibiotics as drug-resistant bacteria already contribute to at least 700,000 deaths globally each year. According to the World Health Organization (WHO), the number of deaths will increase to an alarming 10 million by 2050 if the current trend persist [2].

The etiology of AR is multifactorial, with one of the primary factors being excessive use of antibiotics [1]. According to data from the Danish Integrated Antimicrobial Resistance Monitoring and Research Program (DANMAP, https://www.danmap.org/reports/2023), the prescription of antibiotics in Denmark has increased in periods during recent decades, accompanied by an increase in resistant bacteria during the last decade. Currently, surveillance of AR is predominantly based on analysis of samples from hospitalized individuals [2], which probably does not reflect the background population. Hence, data on AR is most likely influenced by cohort bias, and therefore new methods and strategies for screening for AR are urgently needed to determine the prevalence of AR in the healthy non-hospitalized population.

Surveillance data from the U.S. reported a significant increase in AR among oral streptococci in the period from 2010 to 2020 [3], which is a potential concern as oral streptococci are the most predominant member of the oral microbiota [4]. In addition, a small-scale Swedish study demonstrated that as little as a single dose of 2-gram amoxicillin induced a significant selection of oral streptococci with AR [5], which highlights that AR resistant oral streptococci are effectively enriched during antibiotic treatment. However, further studies on AR of oral streptococci are needed to confirm these findings in a broader context. Furthermore, to be able to systematically investigate AR of oral streptococci, there is a need to develop a laboratory protocol which in a simple and reproducible way facilitates screening of saliva samples for presence of AR in oral streptococci.

The purpose of the present study was therefore to develop the laboratory protocol needed for screening and identification of AR oral streptococci in saliva samples. Subsequently, the aim was to employ this protocol for screening of AR in a pilot population of 100 individuals seeking dental treatment at the Department of Odontology, University of Copenhagen. We tested the hypothesis that saliva samples can be used for detection of AR in the oral microbiota. In addition, we aimed at identifying potential risk factors for AR.

## Methods

### Study design

The present observational cross-sectional study was performed at the Department of Odontology, University of Copenhagen, Copenhagen, Denmark. The study was approved by the regional ethical committee (H-230160030) and reported to the local data authorization of the Faculty of Health and Medical Science (3236959–4242). Prior to participation, participants provided written informed consent in accordance with the Helsinki Declaration.

The recruitment was performed by a random selection of individuals seeking treatment at the Department during the period of data collection (October – December 2023). Inclusion criteria were age >18 yrs and ability and willingness to provide a saliva sample at the time of data collection.

### Questionnaire-based data

Questionnaire-based data was collected as a self-administered web-based questionnaire. The questions were designed based on the WHO Oral Health Surveys Manual to ensure a standardized oral health survey [6]. The survey instrument comprised 16 items, with each of the items having response categories. The questions addressed demographic and socio-economic parameters, i.e. country of origin, level of education, as well as lifestyle parameters, i.e., medication intake, previous antibiotic consumption, smoking, exercise, alcohol and sugar consumption. Finally, self-perceived general health as well as dental health status were also addressed.

### Collection of saliva

Stimulated saliva was collected following a previously described protocol [7] with minor adjustments. In brief, approximately 5 mL of stimulated saliva was collected from each participant using chewing gum. After collection, saliva samples were kept at 4°C for up to 3 hours and thereafter transported to the laboratory, aliquoted and frozen at −80°C until analysis.

### Screening for antibiotic resistant streptococci in saliva

**Figure 1.**
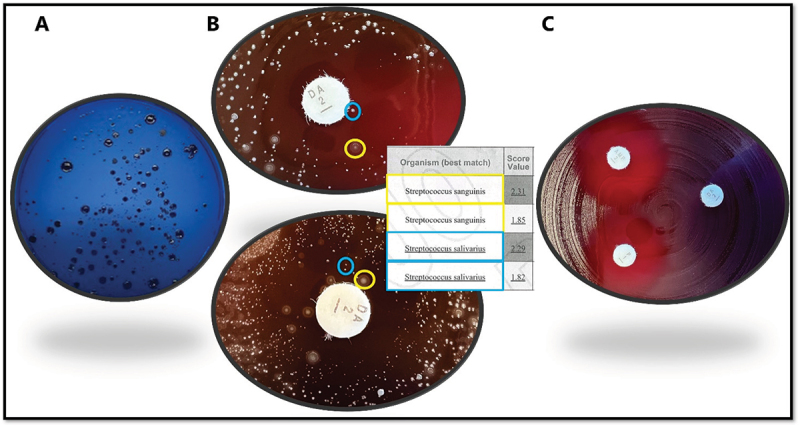
Overview of the laboratory protocol. (A): isolation of *Streptococcus* species using Mitis-Salivarius agar plates with 0.001% tellurite. (B): Screening for antibiotic susceptibility by means of a modified EUCAST disc diffusion strategy using Mueller-Hinton agar plates and antibiotic discs, followed by taxonomic identification of resistant colonies with MALDI-TOF (C): confirmation of AR streptococci strains according to EUCAST standards using the EUCAST disc diffusion method standard procedure.

Saliva samples were thawed and diluted 1000× in sterile 0.9% NaCl. To select for oral streptococci, diluted samples (100 µl) were inoculated on Mitis-Salivarius agar (BD Difco^TM^) plates with 0.001% tellurite (MSAT) and incubated in a 5% CO_2_ atmosphere at 37°C for 48 hours. After incubation, bacterial colonies from agar plates containing at least 100 CFUs were harvested by using a cotton swab, repeated times, to resuspend colony material into a glass tube containing 1 mL 0.9% NaCl. The samples were vortexed vigorously. Next, an antibiotic susceptibility screening was performed using a modified EUCAST disc diffusion strategy (eucast.org), where a dipped cotton swab, was spread evenly on Mueller–Hinton agar comprising 5% defibrinated horse blood and 20 mg/l β-NAD (MH – F agar) plates (SSI Diagnostics, Hillerød, Denmark). Antibiotic discs (penicillin G (1 unit), ampicillin (2 µg) and clindamycin (2 µg) (Oxoid, Thermo Fisher Scientific, UK) were applied, and the plates were incubated in 5% CO_2_ at 37°C for 16–20 hours. Thereafter, the plates were visually inspected, and inhibition zones were read manually using a caliper. Interpretation of resistance towards the respective antibiotics was done according to EUCAST clinical breakpoint tables version 14.0 (http://www.eucast.org/clinical_breakpoints/.) for the viridans group of streptococci. At least two colonies, when possible, of each morphology type or confluent material growing inside or equal to breakpoint values were either directly picked or restreaked to MSAT plates and incubated in 5% CO_2_ at 37°C up to three days. Resulting colonies were identified by means of matrix-assisted laser desorption-ionization time of flight (MALDI-TOF) using a MALDI Biotyper Sirius (Bruker, Billerica, Massachusetts, USA). Isolates identified as streptococcal species were resuspended in 15% glycerol/BHI and frozen at −80°C for resistance confirmation tests (Figure 1).

### Confirmation of antimicrobial susceptibility by EUCAST standard procedure

Confirmation of AR was performed by the disc diffusion method according to EUCAST’s standard procedure with the exception that incubations were performed at 37°C instead of 35 ± 1°C (http://www.eucast.org). In brief, the isolated resistant candidate strains were grown on blood (5%) agar plates for approximately 24 hours. Next, an inoculum was adjusted to McFarland 0.5 following inoculation on 5% MH-F agar plates with antibiotic discs. The 15-15-15 rule was applied, i.e. the inoculum suspension was used within 15 minutes of preparation, discs were applied within 15 minutes of inoculation, and plates were incubated within 15 minutes of disc application. After incubation in 5% CO_2_ at 37°C for 16–20 hours, zones of inhibition were visually inspected and manually evaluated using a caliper. The QC strain *Streptococcus pneumoniae* ATCC 49,619 was added during confirmation tests and AR was interpreted according to EUCAST clinical breakpoint tables (http://www.eucast.org/clinical_breakpoints/). Investigations of the underlying mechanism mediating antibiotic resistance was not conducted, e.g. occurrence of inducible clindamycin resistance (the D phenomenon).

### Statistical analysis

The study population was described using means and standard deviations for continuous variables, and frequencies and percentages for categorical variables. The association between AR and individual background and sociodemographic variables was tested using Pearson’s Chi-squared test or Fisher’s exact test for categorical variables and the Wilcoxon rank-sum test for continuous variables. All analyses were done using R (v. 4.4.1) in RStudio (2024.04.2, Build 764).

## Results

### Demographic and clinical characteristics of the study population

One hundred individuals aged 21–88 years were recruited at the Department of Odontology, University of Copenhagen. The population was comprised of approximately 60% females and with a mean age of 52-years. More than 75% of the study population was born in Europe and had completed secondary education or higher. Approximately 25% of the population had used antibiotics within the past year, and approximately 25% of the population were current smokers. In general, participants perceived their general health and their oral health status as average to excellent (Table 1).

**Table 1.** Study population characteristics by AR.

	Detected AR		
N (%)	No (n = 47)	Yes (n = 43)	P
**Sex**			0.59
*Male*	19 (40)	15 (35)	
*Female*	28 (60)	28 (65)	
**Mean age (sd)**	52.1 (17.9)	52.5 (18.4)	0.81
**Place of birth**			0.70
*Europe*	33 (80)	30 (77)	
*Outside of Europe*	8 (20)	9 (23)	
*Missing*	6	4	
**Highest achieved level of education**			0.47
*No formal schooling or finished 9th grade*	5 (12)	7 (18)	
*Secondary education or higher*	36 (88)	32 (82)	
*Missing*	6	4	
**Received antibiotics within the past year**			0.80
*Yes*	9 (23)	9 (25)	
*No*	31 (78)	27 (75)	
*Missing*	7	7	
**Current smoker**			0.75
*Yes*	9 (23)	9 (26)	
*No*	31 (78)	26 (74)	
*Missing*	7	9	
**Self-rated general health**			0.69
*Poor*	7 (17)	8 (21)	
*Average to excellent*	34 (83)	31 (79)	
*Missing*	6	4	
**Any medical diagnoses**			0.65
*Yes*	23 (50)	19 (43)	
*No*	24 (50)	24 (57)	
**Self-rated oral health**			0.45
*Poor*	11 (28)	7 (20)	
*Average to excellent*	29 (73)	28 (80)	
*Missing*	7	8	
**Last visit to the dentist/dental hygienist**			0.92
*Within the past 12 months*	27 (68)	34 (69)	
*>12 months ago*	13 (33)	11 (31)	
*Missing*	7	8	
**Risk-factor profile**			0.19
*Born outside Europe, highest achieved level of education 9th grade or lower, or received antibiotics within the past year*	12 (30)	16 (44)	
*Missing*	7	7	

P-values for the association between AR and population characteristics were calculated using Pearson’s Chi-squared test or Fisher’s exact test for categorical variables and the Wilcoxon rank-sum test for continuous variables, testing at a 95% significance level. Abbreviations: AR = antibiotic resistance, N = number, sd = standard deviation. Variables include gender, age, place of birth, level of education, antibiotic use within the past year, smoking status, self-rated general and oral health, medical diagnoses, and time since last visit to a dentist or dental hygienist. Additionally, the table compares the risk factor profile of participants, focusing on those born outside Europe, with lower educational attainment, or who received antibiotics in the past year. P-values indicate the significance levels for each comparison.

### Prevalence and distribution of AR in saliva

Saliva samples from ten participants were used as quality control. From a total of the remaining 90 saliva samples, samples from 43 (48%) participants were identified with oral streptococci being resistant to one or more of the tested agents (penicillin, ampicillin or clindamycin). AR against clindamycin was observed in 36 participants, whereas AR towards ampicillin and penicillin was recorded in 15 and 10 participants, respectively. AR against only one of the tested antibiotics was seen in 29 participants, and AR against more than one antibiotic was recorded in 14 participants.

### Species-specific AR patterns

**Figure 2.**
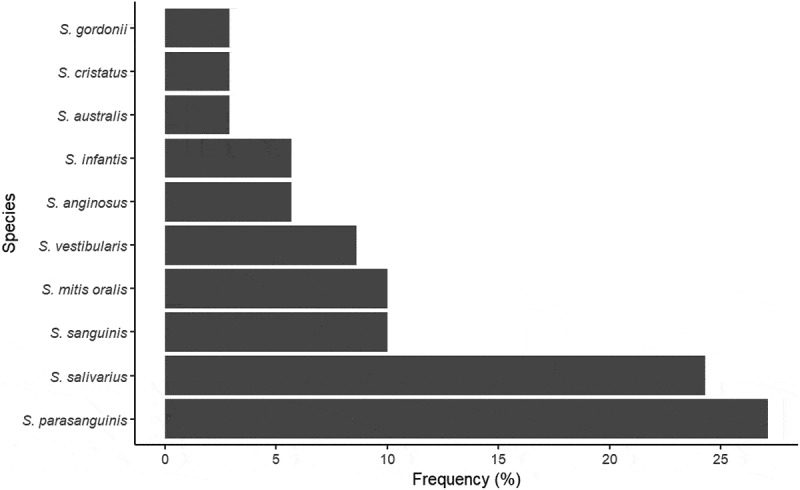
Frequency of resistance by oral *Streptococcus* species. Frequency (%) of antibiotic resistance stratified by the 70 unique *Streptococcus* isolates identified by MALDI-TOF. Note that *S. mitis* and *S. oralis* are not separated by the MALDI-TOF analysis.

A total of 70 unique *Streptococcus* isolates were confirmed as resistant when examining for AR using the standardized EUCAST methodology (listed in Supplementary Table S1). From the AR positive strains, 10 different oral *Streptococcus spp*. were identified, with *S. parasanguinis* and *S. salivarius* being the species most frequently identified, collectively accounting for 51,4% of all cases of resistance (Figure 2). A total of 60,0% of the resistant *Streptococcus* strains were resistant to clindamycin, whereas AR towards penicillin and ampicillin was observed in 17,1% and 22,9% of the strains, respectively.

### Risk factors of AR

None of the registered demographic or sociodemographic parameters registered as well as self-perceived oral and general health was associated with AR, when comparing the group with a positive screening result for AR (*n* = 43 patients), and the group with a negative screening result (*n* = 47 patients). Using a risk-factor profile based on previously studied risk factors for AR (being born outside Europe, having only up to 9th-grade education, or receiving antibiotics in the past year) it was not possible to significantly differentiate the group with AR from the group without AR, albeit there seemed to be a trend in the data (*p* = 0.19) (Table 1). Using data from the risk-factor profile in a post-hoc power-calculation it was estimated that the number of participants needed to be able to detect a significant difference between individuals with and without AR with a 95% significance level and a power of 80% was 186 individuals.

## The developed protocol for screening of AR among *Streptococcus* species in saliva samples

Preliminary experiments showed that other species than streptococci were isolated during the AR screening process, indicating that the initial step using Mitis-Salivarius medium with tellurite was not fully selective. First and foremost, diluting the saliva samples before applying them on MSAT plates was important, see methods, to reduce cases with overgrowth of *Candida* in the resistance zones, obscuring candidate *Streptococcus* AR colonies and impairing isolation. Secondly, picking and growing, on MSAT plates instead of using nonselective medium, after the AR screening, lowered the background of other species. Species other than streptococci identified by MALDI-TOF, during the study, are listed in the Supplementary Table S2.

## Discussion

The present study aimed to uncover the potential of using saliva samples for screening of AR in non-hospitalized adults and to test the use of our newly developed protocol in a pilot population of adult Danes. To the best of our knowledge, this is the first study to develop and validate a standardized laboratory protocol for identification of AR in saliva and to demonstrate the reproducibility and feasibility for saliva-based screening for AR. Hence, data from the present study highlights that saliva samples could be a valuable supplement to current surveillance methodologies for AR, potentially enhancing the ability to screen for AR in the general population during regular dental examinations.

The main finding of the present study in a Danish non-hospitalized population was the high prevalence of AR, which was detected in as much as 48% of the samples (Table 1). This is in concert with a previous study reporting ampicillin resistance of *S. mitis* in 52% of the tested saliva samples from healthy individuals [8], but much higher than a more recent Swedish study which found AR corresponding to streptococci in only 21% of samples in a healthy population [5]. While the consequence of AR is well-known, as demonstrated by the endemic rise in annual AR-related death [9,10], much less is known about the carrier rate of AR resistant strains in the human microbiome, especially in the non-hospitalized part of the population [11]. However, studies from other areas have reported a pronounced presence of AR genes in the microbiome at different body sites such as the skin and the gut [12–14]. Hence, our screening data focusing on presence of AR in oral streptococci reinforces the concern of a widespread presence of AR genes even among healthy individuals. However, future studies with sampling from multiple body sites from the same individuals are needed to evaluate if presence of AR streptococci in saliva is representative of AR in other parts of the human microbiome, as *Streptococcus spp*. do not constitute a significant part of the microbiome at other body sites than the oral cavity. Moreover, it should be noted that in the present study, we did not investigate specific genes for antibiotic resistance, which means that the antibiotic resistance patterns observed could also be a sign of tolerance [15]. Therefore, there is a need for future studies focusing on identification of specific genes in species identified as resistant by use of the current laboratory protocol.

The present study was not able to identify any independent demographic or socioeconomic factors, which could predict the result of the AR screening (Table 1). As demonstrated by a post-hoc analysis of our data, this was due to the sample size of the study, as a sample size greater than 7500 individuals was needed to detect a significant association with our anticipated main exposure, i.e. antibiotic usage during the last 12 months and the risk of a positive screening result of AR in saliva. Indeed, this is in line with contemporary literature, which collectively underscores that while antibiotic consumption is a key driver of AR, the etiology of AR is generally multifactorial [16,17]. Hence, due to the multifactorial nature of AR, we tried to compute a combined risk-factor profile which in addition to antibiotic usage within the last 12 months included two additional risk factors: 1) being born outside Europe and 2) educational level up to 9th-grade, which demonstrated a tendency for identification of individuals with risk of AR (*p* = 0.19). However, a post hoc analysis showed that a sample size of more than 186 individuals was needed to show a significantly different presence of the risk-factor profile in the AR versus the non-AR group. Hence, our preliminary findings from this pilot study align with and expand upon existing research which suggests that AR is driven by a complex interplay of multiple determinants [18,19].

Another interesting finding was the differential expression of AR within different species of genus Streptococcus, with *S. salivarius* and *S. parasanguinis* showing the highest frequency of AR (Figure 2). One explanation for this finding, however, is that *S. salivarius* and *S. parasanguinis* are the two *Streptococcus spp*. with the highest abundance in saliva [20]. While deep odontogenic infections are most often polymicrobial, *Streptococcus* species are often part of the infection [21], but *S. salivarius* and *S. parasanguinis* are not very often identified in oral infections [22,23]. We identified only 4 out of 70 unique AR streptococci as *S. anginosus*, all with clindamycin resistance. This finding is in line with previous studies also reporting that AR of *S. anginosus* towards both penicillin and clindamycin is rare [24,25]. Nevertheless, as streptococci are known by their capacity to perform horizontal gene transfer [26,27], including transfer of AR genes, it is interesting that our findings suggest an uneven distribution of AR with the oral community of *Streptococcus* species. Moreover, we observed a striking difference in prevalence of AR against the three types of antibiotics mostly used by dentist in Denmark and worldwide, i.e. clindamycin, penicillin and amoxicillin (represented as ampicillin) with AR towards clindamycin being identified in 60,0% of all positive strains. The finding of high AR to clindamycin may be the consequence of previous consumption of macrolide antibiotics, whereas the differences in penicillin versus amoxicillin recorded are most likely due to minor differences in zone diameters observed, as illustrated in supplementary table S1. While penicillin, often in combination with metronidazole, is the first choice for dental infections in Denmark, clindamycin is used to treat oral and non-oral streptococcal infections in individuals with penicillin allergy. Hence, this finding may be of particular importance in patients with penicillin allergies.

In the present study, we used saliva samples for screening for AR instead of more traditional types of biological materials, such as blood and feces samples, which are mostly employed in the monitoring of AR. The main advantage of using saliva is the non-invasive sample collection method which in contrast to collection of blood and feces is associated with minimal discomfort [28]. Hence, the high compliance rate allows the method to be applied to almost all population groups and even in rural areas of the world [29]. Additionally, the simple and straightforward collection process doesn’t require specialized personnel, making it a more accessible and cost-effective option for sample collection [30]. Potentially, saliva-based screening could therefore in the future be performed chair-side at the dental office for screening for AR. However, the current challenge is that while sampling of saliva can easily be performed at the dental clinic or the medical doctor’s office, the microbiological method developed and used in the present study requires transfer of the sample material to a microbiological laboratory for analysis performed by specialized personnel. Therefore, there is a need for further development and simplification of the current method for it to be translated into point-of-care use at the dental office [30].

Some limitations apply to the present study, including the limited sample size, which was the natural consequence of the pilot nature of the study design. However, the small sample size was sufficient to demonstrate a high prevalence of AR in saliva from healthy donors. Moreover, the sample size was also sufficient for us to develop and test a novel and reproduceable method that applies EUCAST standards to a complex biological sample, which hopefully will be universally applied by the research community in the future. Another limitation is that we only included three types of antibiotics in our screening protocol, which means that we did not have the possibility to evaluate AR towards a broader spectrum of antibiotics. We deliberately choose the three types of antibiotics, penicillin, amoxicillin and clindamycin, which in accordance with national clinical guidelines are the ones to be used by dentist in Denmark. Hence, we did not include amoxicillin plus clavulanic acid, as this broad-spectrum antibiotic should not be prescribed by general dental practitioners. A final potential limitation is that the analysis was limited to the streptococci part of the oral microbiome. However, multiple studies have shown that genus *Streptococcus* is by far the most abundant in the oral cavity, and therefore most likely representative of AR in the oral microbiome. Moreover, oral streptococci are a major etiological agent of severe oral infections including odontogenic abscesses [21] as well as in potentially life-threatening acute medical conditions, such as infectious endocarditis and brain abscesses [31,32]. Therefore, in our opinion, knowledge of AR in oral streptococci is of critical concern to dentists as well as medical doctors, which is why we deliberately choose to focus our laboratory protocol on analysis of AR in oral streptococci.

## Conclusion

The present study shows that it is possible to use a standardized and reproduceable protocol for non-invasive saliva-based screening for AR in oral streptococci. Moreover, the study highlights a hitherto unprecedented frequency of AR in 48% of saliva samples collected from non-hospitalized adults.

## Supplementary Material

Supplemental Material
