# Supplementary material for: High prevalence of antibiotic resistance of Streptococcus species in saliva from non-hospitalized adults – a pilot study
Source: J Oral Microbiol. 2025 Apr 2;17(1):2486647. doi: 10.1080/20002297.2025.2486647 (PMC11966976; doi:10.1080/20002297.2025.2486647)
Supplement: Supplemental Material [file ZJOM_A_2486647_SM4291.docx]

**Supplementary Table 1.** List of the 70 confirmed AR positive *Streptococcus* species (note that *S. oralis* and *S. mitis* are not separated by MALDI-TOF) with corresponding zone diameters (mm) for respective antibiotics i.e. clindamycin (cm, R<19)), ampicillin (amp, R<15) and penicillin (pen, R<12). Sample ID is indicated (ID). Growth up to the disc i.e. no zone (n.z.).

| **ID** | **Species** | **cm** | **ID** | **Species** | **amp** | **ID** | **Species** | **pen** |
| --- | --- | --- | --- | --- | --- | --- | --- | --- |
| 1 | *S. mitis/oralis* | 16 | 1 | *S. parasanguinis* | 12 | 7 | *S. parasanguinis* | 9 |
| 10 | *S. gordonii* | n.z. | 7 | *S. parasanguinis* | 12 | 18 | *S. vestibularis* | 11 |
| 15 | *S. salivarius* | n.z. | 10 | *S. parasanguinis* | 14 | 34 | *S. parasanguinis* | 10 |
| 18 | *S. cristatus* | n.z. | 15 | *S. salivarius* | 13 | 47 | *S. salivarius* | n.z. |
| 23 | *S. salivarius* | n.z. | 21 | *S. parasanguinis* | 14 | 58 | *S. infantis* | 10 |
| 30 | *S. salivarius* | n.z. | 47 | *S. salivarius* | 11 | 58 | *S. mitis/oralis* | 9 |
| 30 | *S. angionosus* | n.z. | 58 | *S. mitis/oralis* | 13 | 72 | *S. parasanguinis* | 10 |
| 37 | *S. vestibularis* | 15 | 72 | *S. parasanguinis* | 11 | 79 | *S. sanguinis* | 9 |
| 38 | *S. anginosus* | n.z. | 78 | *S. mitis/oralis* | n.z. | 81 | *S. parasanguinis* | n.z. |
| 42 | *S. anginosus* | n.z. | 78 | *S. vestibularis* | 12 | 86 | *S. infantis* | n.z. |
| 47 | *S. salivarius* | n.z. | 79 | *S. parasanguinis* | n.z. | 86 | *S. parasanguinis* | n.z. |
| 50 | *S. parasanguinis* | n.z. | 81 | *S. parasanguinis* | n.z. | 91 | *S. parasanguinis* | n.z. |
| 51 | *S. gordonii* | n.z. | 86 | *S. parasanguinis* | 11 |  |  |  |
| 57 | *S. salivarius* | n.z. | 87 | *S. parasanguinis* | n.z. |  |  |  |
| 59 | *S. salivarius* | n.z. | 92 | *S. parasanguinis* | 8 |  |  |  |
| 60 | *S. sanguinis* | n.z. | 96 | *S. salivarius* | 12 |  |  |  |
| 60 | *S. vestibularis* | n.z. |  |  |  |  |  |  |
| 62 | *S. mitis/oralis* | n.z. |  |  |  |  |  |  |
| 65 | *S. vestibulris* | n.z. |  |  |  |  |  |  |
| 65 | *S. salivarius* | n.z. |  |  |  |  |  |  |
| 66 | *S. parasanguinis* | n.z. |  |  |  |  |  |  |
| 66 | *S. australis* | n.z. |  |  |  |  |  |  |
| 68 | *S. sanguinis* | n.z. |  |  |  |  |  |  |
| 71 | *S. salivarius* | 10 |  |  |  |  |  |  |
| 72 | *S. salivarius* | n.z. |  |  |  |  |  |  |
| 73 | *S. cristatus* | n.z. |  |  |  |  |  |  |
| 76 | *S. australis* | n.z. |  |  |  |  |  |  |
| 76 | *S. vestibularis* | 8 |  |  |  |  |  |  |
| 78 | *S. angionosus* | n.z. |  |  |  |  |  |  |
| 78 | *S. mitis/oralis* | n.z. |  |  |  |  |  |  |
| 80 | *S. salivarius* | n.z. |  |  |  |  |  |  |
| 81 | *S. parasanguinis* | n.z. |  |  |  |  |  |  |
| 82 | *S. infantis* | 13 |  |  |  |  |  |  |
| 86 | *S. infantis* | 15 |  |  |  |  |  |  |
| 88 | *S. sanguinis* | n.z. |  |  |  |  |  |  |
| 90 | *S. salivarius* | n.z. |  |  |  |  |  |  |
| 91 | *S. sanguinis* | n.z. |  |  |  |  |  |  |
| 93 | *S. salivarius* | n.z. |  |  |  |  |  |  |
| 94 | *S. sanguinis* | 18 |  |  |  |  |  |  |
| 96 | *S. mitis/oralis* | n.z. |  |  |  |  |  |  |
| 97 | *S. salivarius* | 16 |  |  |  |  |  |  |
| 99 | *S. sanguinis* | n.z. |  |  |  |  |  |  |

**Supplementary Table 2.** List of species not belonging to streptococci. The strains were isolated during the AR screening procedure and identified by MALDI-TOF. Isolated from antibiotic resistant zone: clindamycin (cm, R<19)), ampicillin (amp, R<15) and penicillin (pen, R<12). Sample ID is indicated (ID).

| **ID** | **Species** | **antibiotic zone** |
| --- | --- | --- |
| 1 | *Rothia mucilaginosa* | cm |
| 5 | *R. mucilaginosa* | cm |
| 7 | *R. mucilaginosa* | cm |
| 18 | *R. mucilaginosa* | cm |
| 22 | *R. mucilaginosa* | cm |
| 24 | *Levilactobacillus brevis*  *Candida albicans* | amp  amp, pen, cm |
| 26 | *Lactobacillus gasseri* | cm |
| 28 | *C. albicans* | amp, pen, cm |
| 33 | *L. plantarum* | pen, cm |
| 34 | *Lacticaseibacillus paracasei*  *L. gasseri* | pen  cm |
| 35 | *R. mucilaginosa* | cm |
| 43 | *L. gasseri* | cm |
| 45 | *R. mucilaginosa* | cm |
| 47 | *R. mucilaginosa* | cm |
| 56 | *Lactobacillus casei* | amp, pen, cm |
| 57 | *Rothia dentocariosa* | cm |
| 58 | *R. mucilaginosa* | cm |
| 63 | *R. dentocariosa* | cm |
| 69 | *Lacticaseibacillus rhamnosus* | cm |
| 70 | *C. albicans* | amp, pen, cm |
| 73 | *Rothia aeria* | cm |
| 80 | *L. casei*  *L. plantarum* | amp  pen |
| 87 | *L. plantarum* | cm |
| 92 | *L. plantarum* | pen |
| 97 | *R. dentocariosa* | cm |
